# Improving Treatment Efficacy of *In Situ* Forming Implants via Concurrent Delivery of Chemotherapeutic and Chemosensitizer

**DOI:** 10.1038/s41598-020-63636-x

**Published:** 2020-04-20

**Authors:** Selva Jeganathan, Emily Budziszewski, Christopher Hernandez, Anshul Dhingra, Agata A. Exner

**Affiliations:** Departments of Biomedical Engineering and Radiology, Case Western Reserve University, 10900 Euclid Ave, Cleveland, OH 44106 USA

**Keywords:** Cancer imaging, Chemotherapy

## Abstract

P-glycoprotein (Pgp), a member of the ATP-binding cassette family, is one of the major causes of multidrug resistance in tumors. Current clinical treatments to overcome MDR involve the co-delivery of a Pgp inhibitor and a chemotherapeutic. A concern for this treatment that has led to varied clinical trial success is the associated systemic toxicities involving endogenous Pgp. Local drug delivery systems, such as *in situ* forming implants (ISFIs), alleviate this problem by delivering a high concentration of the drug directly to the target site without the associated systemic toxicities. ISFIs are polymeric drug solutions that undergo a phase transition upon injection into an aqueous environment to form a solid drug eluting depot allowing for a high initial intratumoral drug concentration. In this study, we have developed an ISFI capable of overcoming the Pgp resistance by co-delivering a chemotherapeutic, Doxorubicin (Dox), with a Pgp inhibitor, either Pluronic P85 or Valspodar (Val). Studies investigated *in vitro* cytotoxicity of Dox when combined with either Pgp inhibitor, effect of the inhibitors on release of Dox from implants in PBS, *in vivo* Dox distribution and retention in a subcutaneous flank colorectal murine tumor, and therapeutic response characterized by tumor growth curves and histopathology. Dox + Val showed a 4-fold reduction in the 50% lethal dose (LD_50_) after 48 hours. Concurrent delivery of Dox and Val showed the greatest difference at 16 days post injection for both Dox penetration and retention. This treatment group had a 5-fold maximum Dox penetration compared to Dox alone ISFIs (0.53 ± 0.22 cm vs 0.11 ± 0.11 cm, respectively, from the center of the ISFI). Additionally, there was a 3-fold increase in normalized total intratumoral Dox intensity with the Dox + Val ISFIs compared to Dox alone ISFIs (0.54 ± 0.11 vs 0.18 ± 0.09, respectively). Dox + Val ISFIs showed a 2-fold reduction in tumor growth and a 27.69% increase in necrosis 20 days post-injection compared to Dox alone ISFIs. These findings demonstrate that co-delivery of Dox and Val via ISFI can avoid systemic toxicity issues seen with clinical Pgp inhibitors.

## Introduction

Annually, approximately 650,000 cancer patients receive neoadjuvant, standard or adjuvant chemotherapy^1^. Systemic chemotherapy is severely hampered by its dose-limiting toxicity, inefficient transport, and limited retention in tumors leading to sub-lethal doses^2^. These sub-lethal concentrations of the drug can cause mutations in cancer cells leading to multidrug-resistant (MDR) tumors. MDR is described as the ability of cancer cells to be resistant to a multitude of drugs that are structurally and functionally different^3^. Numerous mechanisms are involved in the development of MDR^4^. One of the most studied mechanisms of MDR is the upregulation of transmembrane ATP-binding cassette (ABC) transporters, such as P-glycoprotein (Pgp). They function through ATP hydrolysis at the intracellular domain causing a mechanical conformation of the Pgp thus ejecting the drug into the extracellular space^3,5,6^.

Numerous approaches have been developed in order to overcome Pgp mediated MDR in several types of cancers^6–10^. Current clinical trials attempt to systemically deliver a Pgp inhibitor, either synthetic or natural compounds, which modulate the expression and functionality of Pgp^5,11–13^. Pluronic P85 (P85), a tri-block synthetic copolymer, has been extensively studied and has been shown to disrupt ATP production in MDR cells, leading to the reduced functionality of Pgp^13–16^. Site-specific inhibitors, such as Valspodar (Val), competitively bind with the drug binding domain in Pgp and directly inhibit the conformation change required to eject the drug^16–18^. Val has made it to Phase III clinical trials for patients with acute myeloid leukemia but was terminated early due to excessive mortality^19^ and interactions with cytochrome P450 3A4, a liver enzyme, causing poor drug metabolism leading to toxic drug plasma concentrations^20–22^.

Lack of clinical success, mainly due to systemic toxicity, has created a need for better delivery of these Pgp inhibitor. Local drug delivery systems (DDS) have been used in preclinical research for glioblastomas, hepatocellular carcinoma, lung, and abdominal cancers, as an alternative method to deliver high concentrations of drug to the target site without associated systemic toxicities^11,23–27^. However, local DDS struggle with intratumoral drug retention within the target site due to several factors, such as the convective flow from the interstitial fluid/blood vessels, excretion due to Pgp, intracellular drug metabolism, and many more^28–30^. Here, we have developed an *in situ* forming implant (ISFI)^31^ capable of locally delivering a Pgp inhibitor and chemotherapeutic, through a minimally invasive injection procedure using a small-gauge needle. Our delivery system was tested in a murine colorectal cancer (CRC) model. Lack of clinical success are attributed to MDR which occurs in 90% of patients with metastatic CRC^32–34^. This approach can concurrently address the systemic toxicity issues and improve local drug retention within the tumor over time. Upon injection into an aqueous environment (e.g. a tumor), the ISFI will phase invert from a liquid solution into a solid depot, co-releasing a chemotherapeutic, Doxorubicin (Dox), and a Pgp inhibitor, P85 or Val. In this study, we have evaluated the ability of both Pgp inhibitors to improve the Dox penetration and retention intratumorally and  enhance the therapeutic efficacy.

## Methods and Methods

### Materials

Poly(DL-lactic-co-glycolic) (PLGA, acid-capped, 75:25, MW 28.8 kDa, inherent viscosity 0.28 dL/g) was obtained from Evonik Corp (Parsippany, NJ). N-methyl-2-pyrrolidinone (NMP) and Valspodar were obtained from Sigma–Aldrich (St. Louis, Missouri). Dox HCl was obtained from LC Laboratories (Woburn, MA). Pluronic P85 were obtained from BASF (Ludwigshafen, Germany). RPMI-1640, fetal bovine serum, and penicillin-streptomycin were obtained from ThermoFisher Scientific (Waltham, MA). WST-1 was obtained from Roche Applied Sciences (Penzberg, Germany). All items were used as received.

### Tumor cells

Human colorectal carcinoma cells, HCT-15, were chosen due to documented overexpression of Pgp^35^, and were obtained from American Type Culture Collection (Rockville, MD). HCT-15 cells were maintained in RPMI-1640 media supplemented with 10% fetal bovine serum and 1% penicillin-streptomycin in an atmosphere of 5% CO_2_ at 37 °C.

### Cytotoxicity of co-incubation of Dox and Pgp inhibitor

To determine inherent toxicity of each Pgp inhibitor, HCT-15 cells were seeded in a 96 well plates at 5000 cells/well in 200 µL of FBS supplemented media and allowed to reattach overnight. After attachement, the media was replaced with 200 µL of varying Pgp inhibitor concentrations (0 to 100 µg/mL for Val and 0 to 1000 µg/mL for P85 in FBS supplemented media) for 24 and 48 hours. After the exposure time, cells were washed in 1X PBS twice and viability was determined by incubating the cells in 100 µL of WST-1 (1:10 dilution of stock WST-1 in no FBS supplemented RPMI 1640) for 3 hours.

To determine chemosensitization effects, HCT-15 cells were seeded in a 96 well plates at 5000 cells/well in 200 µL FBS supplemented media and allowed to reattach overnight. After attachment, the media was replaced with 200 µL of varying concentration of Dox (0 to 1000 µg/mL) and the highest non-lethal concentration of the Pgp inhibitor seen for 24 and 48 hrs (1 µg/mL for Val and 0.1 µg/mL for P85). After the exposure time, cell viability was determined by washing two times in 1X PBS and incubating cells in 100 µL of WST-1 for 3 hours (1:10 dilution of stock WST-1 in no FBS supplemented RPMI 1640).

Cell viability was calculated by comparing the absorbance of the treatment group to the no treatment group using a plate reader at an absorbance of 450 nm (Tecan Ltd, Infinite 200 series) and displayed as the 50% lethal dose (LD_50_), the amount of Dox required to reduce cell viability to 50%. The resistance reversion index (RRI) was calculated with the following formula:

$$RRI=\,\frac{\,L{D}_{50}\,(Dox)}{L{D}_{50}\,(Dox+Pgp\,inhibitor)},$$

where a larger index value correlates to a better sensitization. Nonlinear regression curve fits ([inhibitor] vs response (three parameters)) were applied to each cell viability curve in Prism 8. These curves reported LD_50_ values for each treatment group which was used to determine the RRI values. Goodness of fit (R^2^) values are reported in each figure.

### ISFI solution preparation

ISFI solutions consisted of PLGA co-dissolved with Dox and a Pgp inhibitor in NMP. Mass ratios of PLGA to NMP to Dox were 39:60:1. P85 and Valspodar were incorporated at 5 wt.% and 1 wt.%, respectively. The addition of the Pgp inhibitor reduced the overall wt.% of the NMP. Dox mass ratio was determined by delivering a clinical dosage to the tumor^36^. Val mass ratio was determined by delivering a third of current phase II and III trials^37,38^. Pluronic mass ratio was determined by delivering similar quantities to the phase II study using Pluronic^39^. The *in vivo* Pgp inhibitor concentration was also equivalent to the concentration used in the *in vitro* cytotoxicity assay. The components of the ISFI solution were added together and allowed to mix overnight inside an incubator shaker at 37 °C. ISFI solutions were used within 24 h of mixing.

### ISFI Dox release

To measure the rate of Dox release, 50 μL of each ISFI were injected into 10 mL of 1X PBS in a glass scintillation vial, capped and placed inside an incubator shaker at 37 °C at 60 rpm. At predetermined time points (t = 2, 4, 6 h and 1, 2, 3, 5, 7, 10 and 14 days), PBS bath-side solution was  collected to measure release. Within the first day, 1 mL of PBS was collected and replaced with 1 mL of fresh PBS. After the first day, 1 mL of PBS was collected, then the rest of the solution was removed and replaced with a fresh 10 mL of PBS. This was done to ensure infinite sink conditions, where the bath-side concentration would be 10 times less than the maximum solubility of Dox in PBS. Cumulative release of drug was determined by measuring the fluorescence intensity of the samples collected and compared to a standard curve using a plate reader at an excitation/emission wavelength of 495/595 nm (Tecan Ltd, Infinite 200 series). The release was normalized to the theoretical 1 wt.% Dox loading prior to injection.

### Subcutaneous HCT-15 mouse tumor model

Studies involving 4-week-old BALB/c nude mice (n=5/group) were received by the Athymic Animal & Xenograft Core and approved by the Case Western Reserve University Institutional Animal Care and Use Committee. All animal experiments were performed in accordance with relevant guidelines and regulations of Case Western Reserve University Institutional Animal Care and Use Committee. Prior to the commencement of experiments the mice were maintained in an aseptic environment for 7 days to acclimate to the new environment. All food, water, and bedding were sterilized before use. HCT-15 cells (5 × 10^6^ cells in 0.1 mL of 1X PBS) were subcutaneously injected into the dorsal side of each mouse. All procedures were performed in a laminar flow hood using aseptic techniques. Tumor growth was monitored daily until the beginning of the study and then on 2, 4, 8, 12, 16, and 20 days after the start of the study.

### Intratumoral Dox distribution and tumor growth study

**Figure 1:**
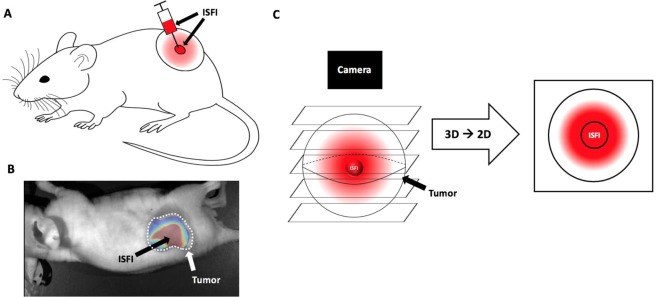
(**A**) Intratumoral ISFI injection into a subcutaneous murine tumor. (**B**) Intratumoral Dox loaded ISFI (red) fluorescent image after masking the background mouse signal. (**C**) Schematic of Dox distribution above and below ISFI being collected into a single pixel value after image acquisition.

Subcutaneous tumors were grown for 10 days then injected with 40 μl of ISFI solution through a 21-gauge needle (Fig. 1A). Mass of the ISFI syringe was measured before and after injection to ensure equivalent ISFI injections. Dox distribution was evaluated 0, 2, 4, 8, 12, 16, and 20 days post-injection with a fluorescent optical imaging system (CRI Maestro, Caliper Life Sciences) with a blue excitation (445–490 nm)/green emission (long pass 580 nm) filter. Total Dox intensity, radial distribution, and max penetration were analyzed using a custom-made MATLAB (Mathworks) script followed by image analysis in ImageJ. Here, intratumoral ISFI images were imported into MATLAB where the image background fluorescence determined from mice with no ISFI injections was removed (Fig. 1B). In ImageJ, a line intensity profile plugin with a line width of 300 was used on the image to measure the signal intensity across the tumor. All points in this profile were added to determine the total Dox intensity in the tumor and normalized to the total intensity post injection^40,41^. Radial distributions were plotted from these intensity profiles where maximum intratumoral Dox penetration of each formulation was determined. Nonlinear regression lines were fitted to each radial distribution using Prism 8. Penetration values were collected above 0.1 normalized intensity to ensure a sufficient signal to noise ratio. It is important to note that Dox distributes radially from the ISFI. Optical images will combine fluorescent intensities above and below the ISFI into a single pixel value as seen in Fig. 1C. Tumor size was monitored every other day. Tumor dimensions were measured using a caliper. and tumor volume was calculated using the following formula:

$$V=\frac{({W}^{2}\times L)}{2},$$

where V = tumor volume, L = length tumor and W = width of tumor^42^.

### Histopathology analysis

After resection, tumors were fixed with paraformaldehyde and frozen in an optical cutting temperature (OCT) compound. Samples were sliced at 7 μm and stained with hematoxylin and eosin. Histological slices were imaged with a microscope slide scanner (Olympus vs120). The necrotic area was calculated using ImageJ by drawing a region of interest around the necrosis area seen in the H&E stain. The necrosis percentage was determined by dividing the necrotic area by the area of the slice.

### Statistical analysis

Statistical analysis was performed using either a student t-test or ANOVA, assuming unequal variances between data sets in Prism 8. Significant differences among means of groups were evaluated using a Tukey multiple comparison test. All data are reported as mean ± standard deviation.

## Results

### Cytotoxicity of co-incubation of Dox and Pgp inhibitor

**Figure 2:**
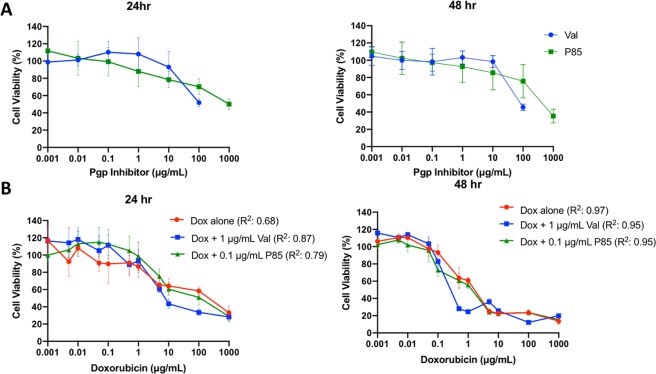
(**A**) Inherent cytotoxicity of Val and P85 incubated at 24 and 48 hrs. (**B**) Chemosensitization cytotoxicity curves of co-incubation varying Dox concentration and non-lethal Pgp inhibitor concentration. Co-incubation of Dox and Val after 48 hours showed a 4-fold reduction in the LD_50_. N = 5 for all samples.

Inherent toxicity curves of HCT-15 cells incubated with either P85 or Val for 24 and 48 hr are shown in Fig. 2A. Nonlethal concentrations (~100% cell viability) determined for Val and P85 for both 24 and 48 hours were 1 *μ*g/mL and 0.1 *μ*g/mL, respectively. These concentrations were then used for the following chemosensitization study. After a 24-hour incubation, Dox + Val showed a 1.33 fold decrease and Dox + P85 showed a 0.57 fold decrease in LD_50_ compared to Dox alone. After a 48-hour incubation, Dox + Val showed a 4 fold decrease and Dox + P85 showed a 1.38 fold decrease in LD_50_ compared to Dox alone. Resistance reversion index (RRI) values are shown in Table 1.

**Table 1:** LD_50_ and resistance reversion index (RRI) values for each treatment.

Treatment	24 hr LD_50_ (*μ*g/mL)	24 hr RRI	48 hr LD_50_ (*μ*g/mL)	48 hr RRI
Dox	3.47	1	0.68	1
Dox + P85	6.05	0.57	0.49	1.38
Dox + Val	2.61	1.33	0.17	4

### ISFI Dox release

**Figure 3:**
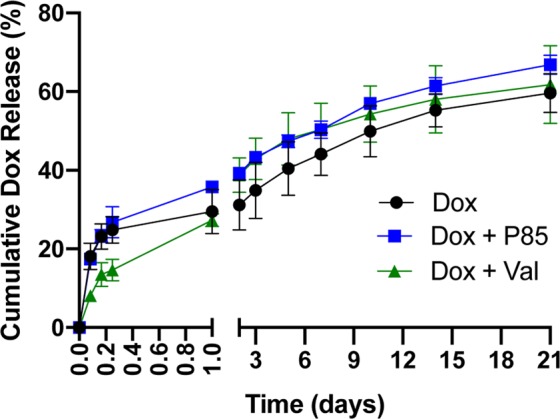
Effects of incorporating a Pgp inhibitor on the release of Dox from the ISFI. No significant differences were seen with the addition of a Pgp inhibitor after 1 day. N = 3 for all samples.

Effects on Dox release from the ISFI with the addition of a inhibitor were evaluated in a PBS dissolution study over the course of 20 days as shown in Fig. 3. Dox + Val ISFIs showed a significant (*p* < 0.05) reduction in the release of Dox compared to Dox alone and Dox + P85 ISFIs up to 6 hours post-injection. Dox, Dox + P85, and Dox + Val ISFIs showed a 29.5 ± 5.6%, 35.8 ± 1.0%, 27.3 ± 1.6%, respectively, release of Dox over the course of 1 day. Over the next 20 days, no significant differences were seen in the release of Dox with the incorporation of either Pgp inhibitor. The average release after 21 days post-injection for Dox, Dox + P85, and Dox + Val ISFIs were 59.61 ± 4.93%, 66.86 ± 2.44%, and 61.75 ± 9.89%, respectively.

### Intratumoral Dox distribution

**Figure 4:**
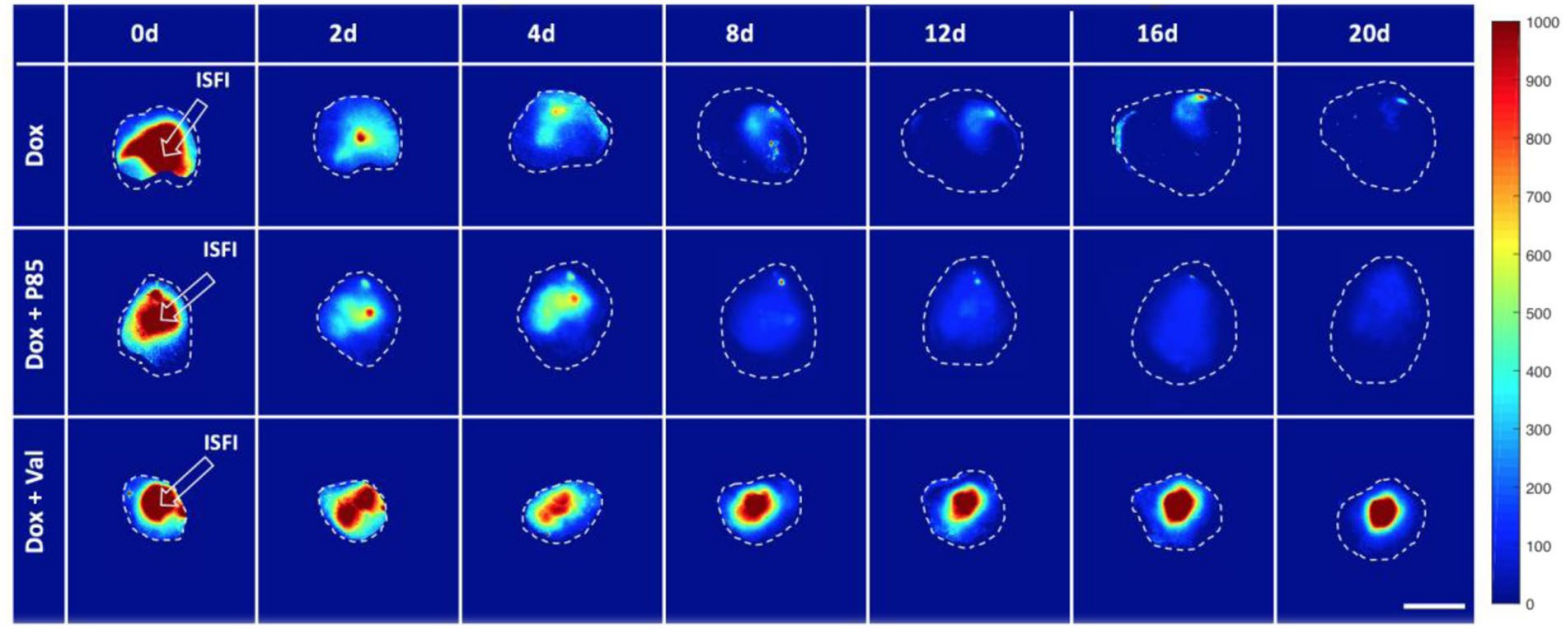
Representative optical fluorescent images of intratumorally injected Dox-loaded ISFIs. Dashed white lines indicate tumor boundaries in each case. Retention of Dox intensity can be seen up to 20 days post-injection for Dox + Val ISFIs. Scale bar = 1 cm. N = 5 for all samples. Fluorescent intensity are in arbitrary units.

**Figure 5:**
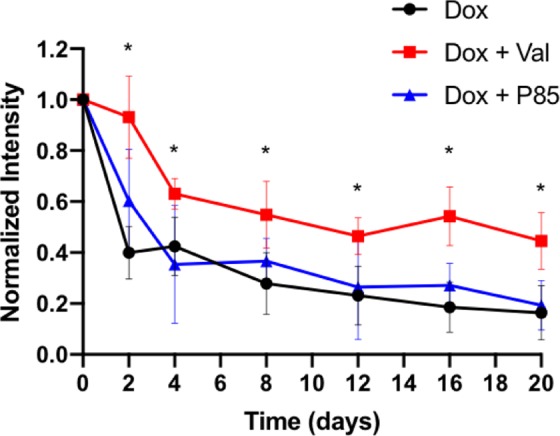
Normalized intratumoral Dox fluorescent intensity over time. Dox + Val ISFIs showed a significant increase in the normalized Dox intensity over time suggesting greater intratumoral Dox retention. *p < 0.05. N = 5 for all samples.

**Figure 6:**
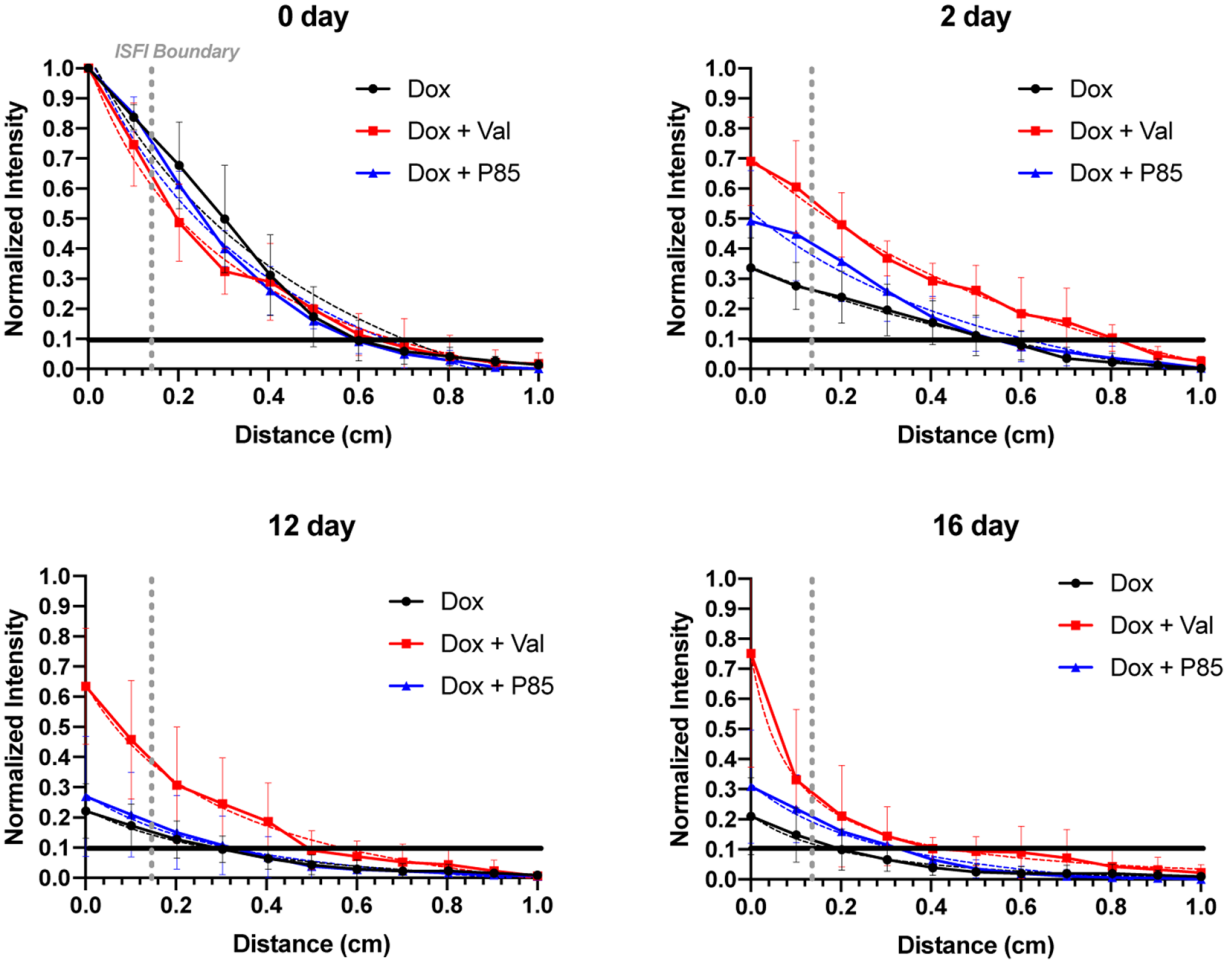
(**A**) Radial distributions of Dox intensities from the center of the intratumoral ISFI over time. Nonlinear regression lines were fitted to each curve and shown as a dashed line. Dox + Val ISFIs showed a consistent increase in radial intensity over time compared to the other groups. Dashed gray lines represent edge of the ISFI. Black lines are placed at 0.1 normalized intensity to ensure sufficient SNR. Maximum Dox penetration values were determined from this 0.1 normalized intensity with respect to distance and shown in Fig. 7. N = 5 for all samples.

**Figure 7:**
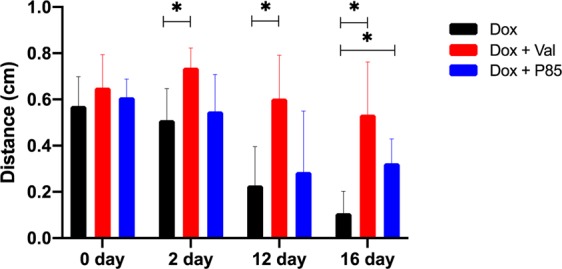
Maximum intratumoral Dox penetration from the center of the ISFI. Dox + Val ISFIs showed a significantly higher Dox retention at further distances from the center of the ISFI over time. *p < 0.05. N = 5 for all samples.

To evaluate the effects of the Pgp inhibitor on Dox distribution within a tumor, 2D fluorescence images of the tumors were collected in mice over 20 days following ISFI injection; representative images are shown in Fig. 4. Total fluorescent intensity per area at each time point is plotted in Fig. 5. Tumors treated with Dox + Val ISFIs showed a significant increase in drug retention compared to tumors treated with Dox alone throughout the 20-day study. The greatest difference can be seen at 16 days with 3-fold increase in normalized intratumoral Dox intensity with in the Dox + Val ISFIs compared to Dox alone ISFIs (0.54 ± 0.11 vs 0.18 ± 0.09, respectively). Radial fluorescence intensity was measured from the center of the ISFI over time and shown in Fig. 6. Post injection, an equivalent release of Dox is seen between all treatment groups. At 2, 12, and 16 days, the Dox + Val ISFIs showed a significant increase in the retention of Dox at distances further from the ISFI center compared to Dox alone. Additional radial distribution data is provided in Supplemental Fig. 2. Figure 7 shows that Dox + Val ISFIs had a significant maximum intratumoral Dox penetration on days 2, 12, and 16 days post injection. The greatest difference was seen at 16 days; Dox + Val ISFIs showed a nearly 5-fold increase in maximum Dox penetration compared to Dox alone ISFIs (0.53 ± 0.22 vs 0.11 ± 0.11 cm, respectively, from the center of the ISFI).

### Subcutaneous tumor growth study

**Figure 8:**
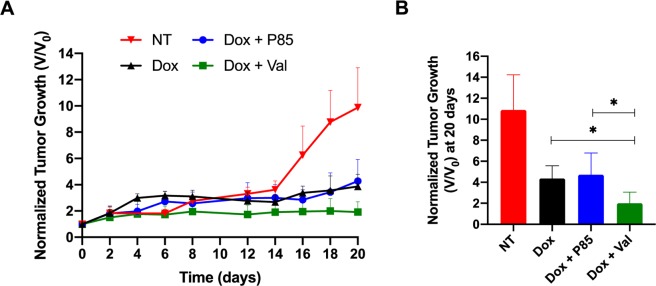
Intratumoral ISFI treatment. (**A**) Tumor volumes measured over time, and (**B**) average tumor volumes for each treatment group at the end of the 20-day study. Dox + Val ISFIs showed a significant inhibition of tumor growth after 20 days. *p < 0.05. N = 4 for NT and N = 5 for all other samples. (NT: no treatment).

Therapeutic efficacy of concurrently delivering a Dox and a Pgp inhibitor from an ISFI was evaluated over 20 days Tumor growth studies (Fig. 8) showed a significant inhibition of tumor growth when co-delivering Dox with Val compared to all other groups. At the end of the 20-day study, tumors treated with Dox + Val ISFIs showed a normalized tumor volume of 2.00 ± 1.06. This was a 2-fold reduction compared to Dox alone and Dox + P85 ISFIs (4.36 ± 1.20 and 4.71 ± 2.07, respectively).

### Histological evaluation

**Figure 9:**
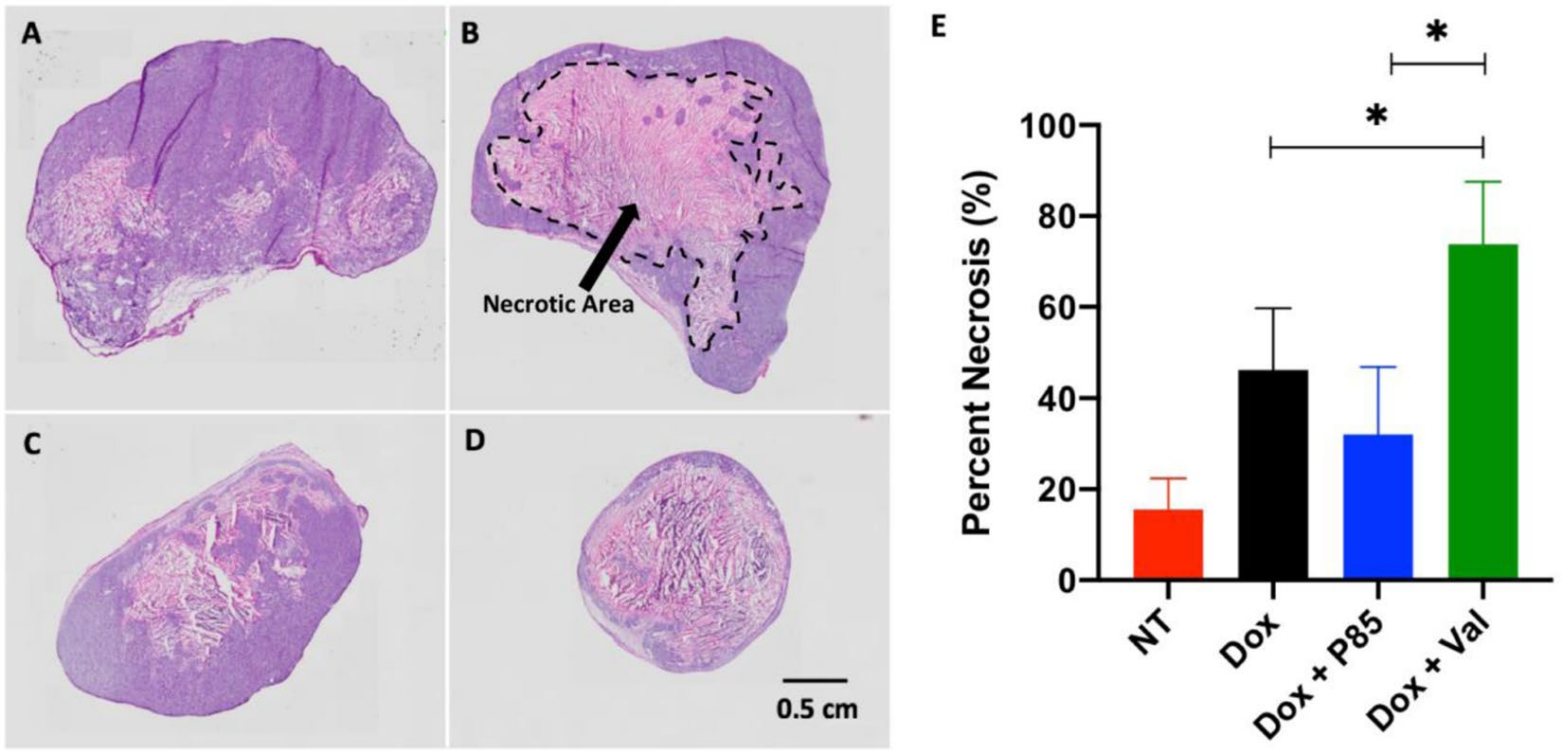
Representative H&E histological images of (**A**) No-treatment (**B**) Dox alone (**C**) Dox + P85 and (**D**) Dox + Val ISFIs. (**E**) Necrosis percentage of all tumor slices. Dox + Val ISFIs showed the greatest necrosis after 20 days. N = 4 for NT and N=5 for all other samples. (NT: no treatment). Necrotic area is shown in a dashed black line in (**B**).

In order to assess the degree of necrosis, H&E staining was performed for each treatment group. Representative H&E slices are shown in Fig. 9A–D and calculated necrosis percentage is seen in Fig. 9E. As expected, the NT group showed the lowest necrosis percentage at 15.53 ± 6.83%. Interestingly, tumors treated with Dox alone showed greater necrosis compared to tumors treated with Dox + P85 ISFIs (46.13 ± 13.55% vs 32.07 ± 14.72%, respectively). Treatment with Dox + Val ISFIs showed the greatest amount of necrosis (73.82 ± 13.73%).

## Discussion

A single non-lethal exposure to chemotherapeutics can lead to MDR, where one of the best-understood mechanisms is the upregulation of Pgp drug efflux transporters that presents itself on the tumor cell membrane^43^. Pgp effluxes chemotherapeutics from the cytoplasm causing sublethal concentrations within the tumor cell^12,44,45^. Current treatments in clinical trials include delivering competitive inhibitors, such as Val^37,38^, concurrently with the chemotherapeutic. However, these treatments suffer from disruptions to drug metabolism, toxic drug plasma concentrations, and interactions with endogenous Pgp^37,38,46^. Local drug delivery systems, such as ISFIs, can deliver high concentrations of the drug directly to the tumor site, thus avoiding systemic toxicities seen in clinical Pgp inhibitors^47^. In this study we locally co-delivered Dox and a Pgp inhibitor that led to a greater retention of the Dox within the tumor mass and enhanced the therapeutic outcomes.

Chemosensitization effects were investigated by co-incubating Dox and a Pgp inhibitor with HCT-15 cells, which have been shown to overexpress Pgp^48^. The co-incubation of Dox with either Val or P85 showed an enhanced reduction in the LD_50_ as seen with a high RRI value. Pluronic P85, a tri-block copolymer composed of polyethylene oxide (PEO) and polypropylene oxide (PPO) in the ratio of PEO_26_-PPO_40_-PEO_26_, has been studied extensively. It has been demonstrated to sensitize the cell through disruption in mitochondrial ATP activity by inhibition of the electron transport chain^13,15^. This decrease in ATP production reduces the efficacy of Pgp, an ATP-dependent efflux transporter, allowing for the enhanced cellular accumulation of Dox enhancing the cytotoxicity. Val has been characterized as a competitive substrate inhibitor of Pgp, where it complexes with the drug binding domains of Pgp inhibiting the mechanical conformational change required to eject the drug^6,16,20,45^. This inhibition allows for increased intracellular concentration of Dox leading to greater cytotoxicity.

Dox release studies showed that the addition of Val to the implants resulted in a significantly smaller burst release of Dox within 6 hours, which may be due to Val’s high lipophilicity altering the solvent/non-solvent exchange kinetics^49^. These reports are consistent with other studies, where the addition of lipophilic molecules into ISFIs, such as glycerol monostearate, ethyl heptanoate, stearic acid, ethyl heptanoate, methyl heptanoate, and ethyl nonoate, reduce solvent excretion therefore leading to a reduction in burst release^50–53^. Dox + P85 ISFIs showed no effect on the burst release, which was equivalent to a previous study^54^. During the diffusion phase where the ISFI is completely solidified, the Dox + P85 and Dox+ Val ISFIs showed no significant differences compared to Dox ISFI. These studies show that although Val has an effect on the burst release of ISFI, the overall release (21 days) of Dox was equivalent between all groups.

Optical fluorescent images of the intratumorally injected Dox + Val ISFIs showed a greater retention of Dox throughout the 20-day study which suggests a greater concentration of Dox intratumorally. Radial distribution profiles of Dox + Val ISFIs showed a greater penetration of Dox from the center of the ISFI over the course of the study. These profiles were used to determine the maximum Dox penetration, where Dox + Val ISFIs showed a significant increase in maximum Dox penetration over time. This may be due to the lower molecular weight of Val (1214 kDa) compared to P85 (4600 kDa), which would allow greater diffusion of Val into more distal tumor cells to elicit a greater retention of the distally penetrated Dox^55,56^.

Tumor volume measurements and histopathology analysis were performed to evaluate the therapeutic efficacy of a single treatment of co-delivering Dox with a Pgp inhibitor. As expected, all treatment groups showed a significant reduction in tumor growth over 20 days compared to the no-treatment group. Tumors treated with Dox + Val ISFIs showed the greatest inhibition of tumor growth, where a two-fold decrease was seen compared to tumors treated with either Dox alone or Dox + P85 ISFIs. This was validated with histological analysis showing a greater necrosis percentage for the Dox + Val ISFIs. The improvement in therapeutic efficacy of the ISFIs loaded with a Pgp inhibitor correlates well with the optical intratumoral Dox images. A greater maximum penetration and total intratumoral retention of Dox seen with the Dox + Val ISFIs suggests there is a larger area of treatment leading to a better therapeutic outcome.

A limitation to the current work is that the treatment only consisted of a single injection of ISFI at the beginning of the study. As shown with the release curves, approximately 50% of the Dox is released within 10 days and another 10% in the following 10 days^22,31,57–59^. Allowing the study to progress further than 20 days and using multiple injections of the ISFI will likely elicit stronger therapeutic effects. This approach as well as incorporation of varying doses of Pgp inhibitors into the ISFIs and examining their distribution within tumor tissue will be the subject of future studies.

## Conclusions

MDR tumors that express Pgp have poor treatment responses due to required elevated drug concentrations to reach therapeutic levels. Current clinical trials using Pgp inhibitors suffer due to systemic toxicity. ISFIs provide an excellent alternative to deliver elevated concentrations of both the chemotherapeutic and Pgp inhibitor bypassing the associated systemic toxicities seen in clinical trials. In this study, Dox + Val ISFIs were able to demonstrate enhanced distribution and retention of the drug leading to a reduced tumor growth and increased necrosis compared to Dox alone ISFIs. This concurrent local delivery of a chemotherapeutic and Pgp inhibitor serves as a promising approach to treat MDR tumors without the toxicities seen with the systemic chemotherapy approach.

## Supplementary information

Supplementary information.
