# Supplementary material for: Improving Treatment Efficacy of In Situ Forming Implants via Concurrent Delivery of Chemotherapeutic and Chemosensitizer
Source: Sci Rep. 2020 Apr 20;10:6587. doi: 10.1038/s41598-020-63636-x (PMC7170888; doi:10.1038/s41598-020-63636-x)
Supplement: Supplementary file 1 — Supplementary information. [file 41598_2020_63636_MOESM1_ESM.pdf]

**Improving Treatment Efficacy of *In Situ* Forming Implants via Concurrent Delivery of Chemotherapeutic and Chemosensitizer**

Selva Jeganathan[1], Emily Budziszewski[1], Christopher Hernandez[1], Anshul Dhingra[1], and Agata A. Exner[1,2]

Departments of Biomedical Engineering[1] and Radiology[2], Case Western Reserve University, 11100 Euclid Ave, Cleveland, OH, 44106 USA

## Materials/Methods:

*Tumor cells:*

Human colorectal carcinoma cells, HCT-15, were chosen due to overexpression of Pgp[1], and obtained from American Type Culture Collection (Rockville, MD). HCT-15 cells were maintained in RPMI-1640 media supplemented with 10% fetal bovine serum and 1% penicillin-streptomycin in an atmosphere of 5 % $CO_2$ at 37 °C.

*Inhibitory Potential Assay:*

To determine the transport function inhibitory potential of the Pgp inhibitor after release from the in situ forming implants (ISFI), HCT-15 cells were seeded in a 96 well plates at 5000 cells/well in 200 µL of FBS supplemented media and allowed to reattach overnight. ISFIs including the Pgp inhibitor and Dox were formulated (description in manuscript) and allowed to mix overnight. 50 $\mu$L of the ISFI solution was injected into 10 mL of RPMI 1640 media supplemented with 10% fetal bovine serum and 1% penicillin-streptomycin. After 6 hours, 200 $\mu$L of the RPMI 1640 media for each sample was incubated with the HCT-15 cells. After 24 hours, cell viability was determined by washing two times in 1X PBS and incubating cells in 100 µL of WST-1 for 3 hours (1:10 dilution of stock WST-1 in no FBS supplemented RPMI 1640).

*Radial Intratumoral Dox Distribution:*

After optical imaging of tumors, images were exported to MATLAB and ImageJ for post image processing. Briefly, background fluorescence from each mouse was removed. The images were

exported into ImageJ to use the intensity profile plugin to generate radial profile intensities. Nonlinear regression lines were fitted to these radial profile intensities in Prism 8. Penetration values were collected above 0.1 normalized intensity to ensure a sufficient signal to noise ratio. The values collected from these nonlinear regression curves are plotted in Table 1.

## Results:

*Inhibitory Potential Assay:*

The activity of both Pgp inhibitors, P85 and Val, in conjunction with Dox after co-release from the ISFIs is shown in Figure 1. Dox alone, Dox + P85, and Dox + Val showed a cell viability of 51.9 ± 8.9%, 35.1 ± 5.3%, 31.7 ± 6.8%, respectively. These findings demonstrate that Pgp inhibitors released from the ISFI retain their bioactivity and capable of enhancing cytotoxicity.

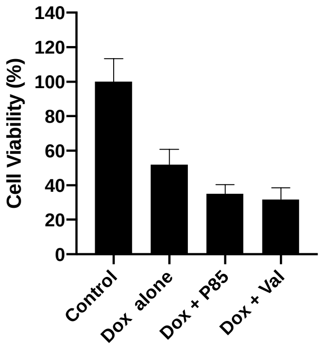


**Figure S1:** Cytotoxicity inhibitory potential of Dox and Pgp inhibitor released form ISFIs.

*Radial Intratumoral Dox Distribution:*

Results in Figure 7 of manuscript show maximum Dox penetration calculated from the radial distribution profiles (solid lines) in Figure 6. Figure S2 show the radial Dox distribution for 4, 8 and 20 days. The dashed lines in Figure 6 are the nonlinear regression curve fits evaluated in Prism 8. The maximum Dox penetration using these nonlinear regression curve fits for 0, 2, 12, and 16 days are shown in Table S1.

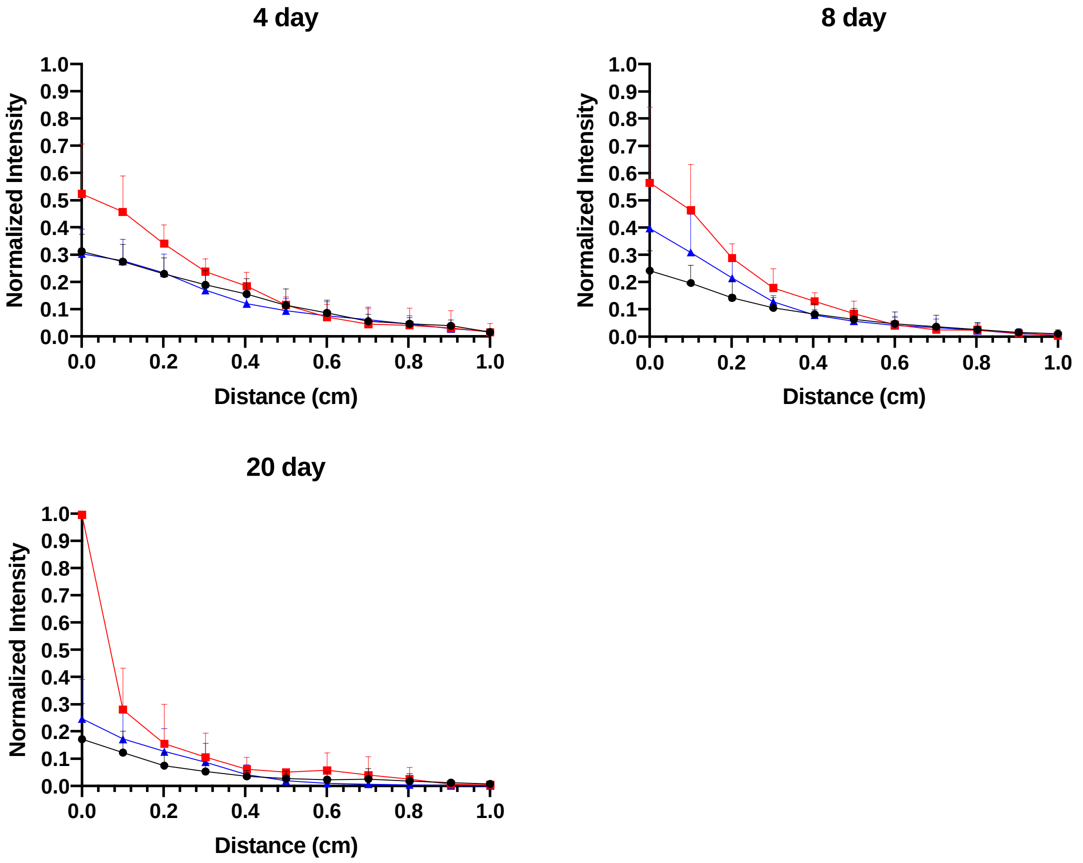


**Figure S2:** All radial distributions of Dox from the center of the intratumoral ISFI over time. Dox + Val group showed an increased radial intensity over time compared to the other groups.

**Table S1:** Dox penetration with 0.1 normalized intensity calculated from nonlinear regression lines.

| Day | Treatment | Distance @ 0.1 Normalized Intensity (cm) |
|---|---|---|
| **0** | Dox alone | 0.69 |
| | Dox + Val | 0.67 |
| | Dox + P85 | 0.66 |
| **2** | Dox alone | 0.54 |
| | Dox + Val | 0.79 |
| | Dox + P85 | 0.60 |
| **12** | Dox alone | 0.27 |
| | Dox + Val | 0.56 |
| | Dox + P85 | 0.31 |
| **16** | Dox alone | 0.18 |
| | Dox + Val | 0.44 |
| | Dox + P85 | 0.32 |

## References

(1) Lee, J. S.; Paull, K.; Alvarez, M.; Hose, C.; Monks, A.; Grever, M.; Fojo, A. T.; Bates, S. E. Rhodamine Efflux Patterns Predict P-Glycoprotein Substrates in the National Cancer Institute Drug Screen. *Mol. Pharmacol.* **1994**, *46* (4).
